# Ameliorative Effect of a Neoteric Regimen of Catechin plus Cetirizine on Ovalbumin-Induced Allergic Rhinitis in Rats

**DOI:** 10.3390/life12060820

**Published:** 2022-05-31

**Authors:** Mohamed A. Morsy, Snehal S. Patel, Anita Bakrania, Mahmoud Kandeel, Anroop B. Nair, Jigar N. Shah, Sabah H. Akrawi, Mahmoud El-Daly

**Affiliations:** 1 Department of Pharmaceutical Sciences, College of Clinical Pharmacy, King Faisal University, Al-Ahsa 31982, Saudi Arabia; anair@kfu.edu.sa (A.B.N.); sakrawi@kfu.edu.sa (S.H.A.); 2 Department of Pharmacology, Faculty of Medicine, Minia University, El-Minia 61511, Egypt; 3 Department of Pharmacology, Institute of Pharmacy, Nirma University, Ahmedabad 382481, Gujarat, India; anitabakrania@gmail.com; 4 Department of Biomedical Sciences, College of Veterinary Medicine, King Faisal University, Al-Ahsa 31982, Saudi Arabia; mkandeel@kfu.edu.sa; 5 Department of Pharmacology, Faculty of Veterinary Medicine, Kafrelsheikh University, Kafr El-Sheikh 33516, Egypt; 6 Department of Pharmaceutics, Institute of Pharmacy, Nirma University, Ahmedabad 382481, Gujarat, India; jigsh12@gmail.com; 7 Department of Pharmacology & Toxicology, Faculty of Pharmacy, Minia University, El-Minia 61511, Egypt; eldaly_m@mu.edu.eg

**Keywords:** allergic rhinitis, antihistamine, catechin, cetirizine, histidine decarboxylase inhibitor

## Abstract

Allergic rhinitis (AR) affects 20–50% of the global population. Available treatments are limited by their adverse effects. We investigated the anti-allergic effects of catechin alone and combined with cetirizine against ovalbumin-induced AR. Rats were sensitized with ovalbumin and received catechin (14 days) and then challenged with aerosolized ovalbumin (1%) to determine AR clinical scores. Histamine, histamine release, and histidine decarboxylase (HDC) activity were determined in blood, peritoneal mast cells, and stomachs, respectively. Vascular permeability and safety were assessed using Evans blue leakage and barbiturate-induced sleeping-time assays, respectively. Catechin and cetirizine binding with HDC was investigated by docking and binding energy analyses. The clinical scores of the combination regimen were superior to either drug alone. All treatments reduced vascular leakage, with no effect on barbiturate-induced sleeping time. Only the catechin-treated rats showed reduced histamine levels and HDC activity. Docking studies revealed that catechin has a 1.34-fold higher extra-precision docking score than *L*-histidine. The binding energy scores for catechin-HDC, *L*-histidine-HDC, and histamine-HDC were −50.86, −37.64, and −32.27 kcal/mol, respectively. The binding pattern of catechin was comparable to the standard HDC inhibitor, histidine methyl ester, but with higher binding free energy. Catechin binds the catalytic residue S354, unlike cetirizine. The anti-allergic effects of catechin can be explained by HDC inhibition and possible antihistaminic activity.

## 1. Introduction

Allergic rhinitis (AR) is one of the most commonly encountered diseases affecting adults, with global estimates ranging from 20–50% of the population [1,2]. AR is associated with deterioration of quality of life, increased loss of work and school days, and impairment of sleep [3,4]. The pathophysiology of AR involves the activation of an inflammation cascade that is initiated by mast cell activation succeeding IgE cross-linking by an allergen. This action triggers the degranulation of mast cells and the release of preformed mediators such as histamine [5,6]. Histamine is a vital mediator that is released during the immediate allergic response from cytoplasmic granules in tissue mast cells and during the late phase response chiefly from recruited basophils. The rapidly synthesized mediators trigger the early symptoms of AR: sneezing, pruritus, and rhinorrhea [4,5,6,7,8].

Histidine decarboxylase (HDC) is the rate-limiting enzyme in the histamine synthesis pathways because it converts histidine to histamine in mast cells and other histamine-producing cells. Thus, HDC serves as an important therapeutic target in allergic diseases [9,10]. The HDC inhibitors are henceforth thought to be beneficial through the reduction of potentially damaging histamine-related local immune responses in allergic diseases [11,12]. The current treatment options available for AR include antihistamines, mast cell stabilizers, non-steroidal anti-inflammatory drugs, corticosteroids, and immunosuppressants. Most of these drugs act immediately to relieve the symptoms of allergic reactions, but usually require multiple dosing and cause central nervous system (CNS) and cardiovascular system adverse effects [13,14,15,16].

Catechin, a flavanol present in several plants including green tea, is an HDC inhibitor [12,17] that can target allergic conditions. Furthermore, the HDC inhibitory effect of epigallocatechin gallate, a closely related catechin derivative, is well established [18,19]. Cetirizine, on the other hand, is a second-generation antihistaminic drug that acts by blocking histamine H_1_ receptors and is used in the management of various allergic conditions [14,15].

This study tests the possible additive effects of cetirizine and catechin as an antiallergic combination for the management of AR. As cetirizine is a histaminergic receptor blocker while catechin is an HDC enzyme inhibitor, their combination is expected to provide the advantages of quick onset (cetirizine) and long-term effects due to inhibition of HDC enzyme by catechin. Therefore, the combination of catechin with cetirizine has been explored as a possible treatment regimen for AR in order to obtain high selectivity along with an absence of CNS side effects [12,20]. Besides, the molecular basis of catechin-induced HDC inhibition was investigated by molecular docking and binding energy calculation.

## 2. Materials and Methods

### 2.1. Chemicals

Catechin, cetirizine, ovalbumin, histamine hydrochloride, compound 48/80 (a potent histamine liberator), and phenobarbitone were purchased from Sigma-Aldrich/Merck KGaA (Darmstadt, Germany). *o*-phthalaldehyde, *L*-histidine, and other chemicals were of analytical grade and were obtained from commercial sources.

### 2.2. Animals and Experimental Protocol

Male Sprague Dawley rats (250–300 g) and male Balb/c mice (25–30 g) were used after 1 week for proper acclimatization to the animal house conditions (24 ± 2 °C temperature, 55 ± 5% relative humidity, and 12 h light/dark cycle). All experiments and protocols described in the current study were approved by the Institutional Animal Ethics Committee (IAEC) of the Institute of Pharmacy, Nirma University (IPS/PCOL/MPH12-13/1009).

Rats were randomized into 5 groups: normal control group, diseased control group, diseased and catechin-treated (100 mg/kg, p.o.), diseased and cetirizine-treated (10 mg/kg, p.o.), diseased and catechin- and cetirizine-treated (50 mg/kg and 5 mg/kg, respectively). A fourteen-day sensitization protocol was carried out by i.p. injection of 100 µg ovalbumin adsorbed on 20 mg alum and dissolved in saline as previously described [12]. Catechin treatment commenced on day 1 and continued daily for 14 days, while cetirizine was administered once on the day of the experiment itself. At the end of the treatment period, animals were challenged with a nasal spray of 1% ovalbumin. Animals were monitored for sneezing, itching, and nasal discharge. Clinical scores of such parameters were calculated based on their frequency and severity (Table 1).

### 2.3. Vascular Permeability Assay

The animals were anesthetized by a mixture of 50 mg/kg ketamine hydrochloride and 10 mg/kg xylazine hydrochloride given intraperitoneally. Animals were turned to the supine position and were fixed to the surgical table. The Evans blue dye leakage method was used for the evaluation of vascular permeability. Evans blue was injected into the jugular vein followed by cannulation in the nasopharynx connected to an infusion pump with phosphate-buffered saline (PBS). Perfusion was carried out in 3 different phases as follows: (1) PBS for 10 min, (2) ovalbumin (0.3% *w*/*v*) for 10 min, and (3) PBS was again perfused continuously for 40 min. Thus, perfusion was continued for a total of 60 min, while the perfusate was collected at 10 min intervals denoted as periods 1 to 6. The centrifuged perfusate samples were then subjected to spectrometric measurements of the dye leakage.

### 2.4. Determination of Histamine Level and HDC Activity

Estimation of mast cell histamine content was determined by isolation of mast cells from the peritoneal cavity after i.p. PBS injection and thorough massage. The fluid collected was then centrifuged and the pellet was resuspended in PBS. Mast cell degranulation by compound 48/80 was followed by an estimation of the histamine content. Spectrofluorometric determination of histamine concentration followed its reaction with *o*-phthalaldehyde to yield a fluorescent conjugate (emission max at 450 nm, excitation at 360 nm). Blood histamine content was measured by an analogous procedure using perchloric acid to release histamine from basophils followed by histamine extraction and treatment with *o*-phthalaldehyde to generate fluorescence measured at the same wavelength stated previously.

HDC assay was performed using the stomach homogenate treated with 1 µg/mL pyridoxal-5′-phosphate and 1 µg/mL dithiothreitol followed by centrifugation and the reaction was started by adding 0.1 µg/mL *L*-histidine. The reaction was terminated by adding HClO_4_, and the formed histamine was measured fluorometrically after reaction with *o*-phthalaldehyde as previously described [12].

### 2.5. Barbiturate-Induced Sleeping Time

The effect of different treatments on the barbiturate-induced sleeping time in Balb/c mice was used to evaluate the CNS safety of catechin, cetirizine, or their combination. Mice in this experiment were divided into four groups: normal control, catechin-treated (100 mg/kg/day, p.o.), cetirizine-treated (10 mg/kg, p.o.), and combination-treated (catechin 50 mg/kg plus cetirizine 5 mg/kg, p.o.). In this experiment, catechin administration was continued daily for 14 days, while the animals received cetirizine only once on the 14th day. On the 14th experimental day, animals received phenobarbitone sodium (45 mg/kg, i.p.). The onset and duration of sleep for each mouse were recorded, and the criterion for sleep was the loss of righting reflex.

### 2.6. Molecular Docking

To check the underlying mechanisms of HDC inhibition by catechin, molecular modeling and docking studies were performed.

#### 2.6.1. Compounds Retrieval and Preparation

The compounds used in the docking study comprised catechin, *L*-histidine, histamine, cetirizine, and histidine methyl ester, and were retrieved from the PubChem database. The LigPrep module of the Maestro package (Schrödinger, New York, NY, USA) was used to desalt and 3D optimize the chemical structures at neutral pH.

#### 2.6.2. HDC Structure Retrieval, Preparation, and Docking

Human HDC was retrieved from the Protein Data Bank (PDB ID 4E1O). The structure comprises human HDC bound with histidine methyl ester with pyridoxal-5′-phosphate. Grid generation and docking run were carried out as previously described [21,22], with slight modifications. The structure-bound ligand was taken as the center for grid generation. During the preparation of the grid, an excluded volume was subtracted from the docking grid. This comprises the volume occupied by pyridoxal-5′-phosphate which is important for the decarboxylation activity of HDC (Supplementary Figure S1). Docking was performed by the extra-precision (XP) docking module. The docking accuracy was confirmed by redocking of the bound ligand and checking the route mean square deviations.

#### 2.6.3. Binding Energy Calculations

The combination of docking and binding energy calculations can give a better insight into the strength of ligand binding to a biological system. Therefore, the binding energies of the compounds with HDC were evaluated using the molecular mechanics-generalized Born surface area (MM/GBSA) approach. The MM/GBSA binding free energies were estimated as follows:

Δ*G_binding_* = *G_complex_* − (*G_drugs_* + *G_PLpro_*) (1)

where the energy term (*G*) is estimated as:

*G* = *E_vdw_* + *E_ele_* + *G_GB_* + *G_SA_* (2)

with *E_vdw_*, *E_ele_*, *G_GB_*, and *G_SA_* as the van der Waals, electrostatic, generalized Born solvation, and surface area energies, respectively. Entropy contributions were not considered in this study.

#### 2.6.4. Binding-Energy Decomposition

Decomposition of the average MM/GBSA binding energy discloses the nature of dominant interactions drugs with HDC.

### 2.7. Statistical Analysis

The data represent the mean ± SEM of six observations. Statistical analysis was performed by GraphPad Prism 7 (GraphPad Software Inc., San Diego, CA, USA). Statistical differences between the means of various groups were evaluated using one-way analysis of variance (ANOVA) followed by Tukey’s test. Vascular permeability data were analyzed using two-way ANOVA followed by Tukey’s test. Data were considered statistically significant at *p* < 0.05.

## 3. Results

### 3.1. Effect of Different Treatment Regimens on AR Clinical Scores

**Figure 1:**
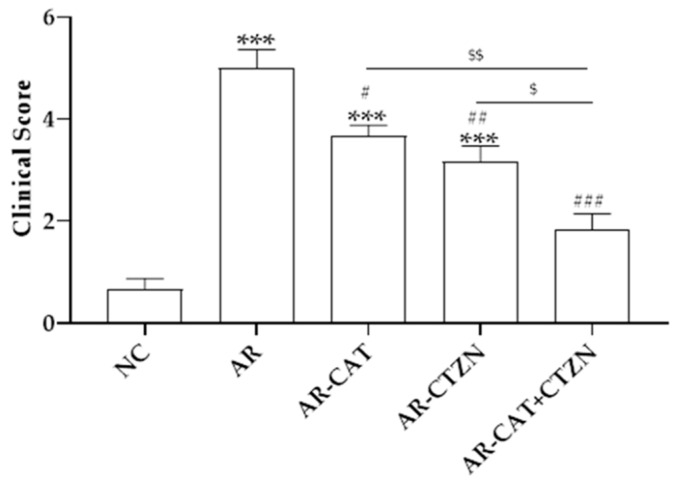
Clinical scoring in allergic rhinitis (AR). Data represent the mean ± SEM of six animals. NC: Normal control, AR: AR disease control, AR-CAT: AR treated with catechin (100 mg/kg/day), AR-CTZN: AR treated with cetirizine (10 mg/kg, p.o.), AR-CAT+CTZN: AR treated with a combination of catechin (50 mg/kg/day, p.o) and cetirizine (5 mg/kg, p.o). ***: significantly different from the NC group at *p* < 0.001. #, ##, ###: significantly different from the AR group at *p* < 0.05, *p* < 0.01, and *p* < 0.001, respectively. $, $$: significantly different from the AR-CAT+CTZN group at *p* < 0.05, *p* < 0.01, respectively.

Ovalbumin provocation at the end of the 14-day sensitization period significantly increased the clinical scores in the AR disease control group as compared with the control animals. Treatment of AR animals with catechin, cetirizine, or their combination significantly reduced the clinical scores when compared with the untreated disease control group. The combination regimen was the most effective in reducing the disease-related clinical scores, which were not statistically significant than those observed in normal control animals (Figure 1).

### 3.2. Effect of Different Treatment Regimens on Vascular Permeability

**Figure 2:**
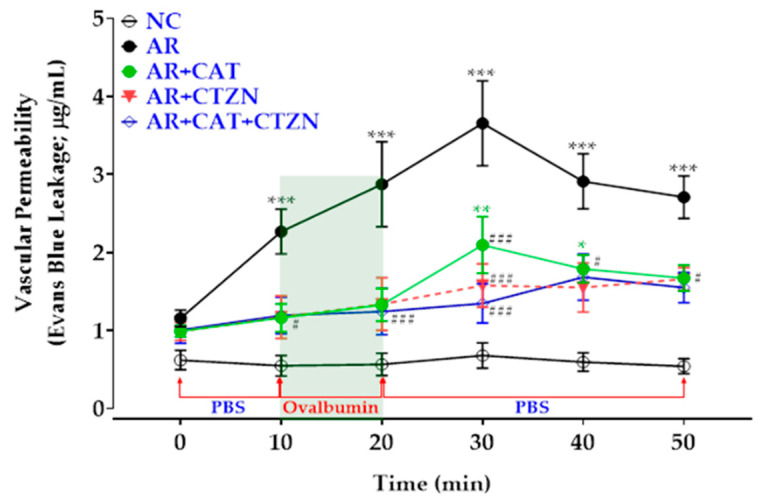
Vascular permeability curve in allergic rhinitis (AR). PBS: phosphate-buffered saline; marks the periods when the nasal cavity was perfused with PBS. Ovalbumin denotes the period of ovalbumin (0.3% *w*/*v*) perfusion. Data represent the mean ± SEM of six animals. NC: normal control, AR: AR disease control, AR-CAT: AR treated with catechin (100 mg/kg/day), AR-CTZN: AR treated with cetirizine (10 mg/kg, p.o.), AR-CAT+CTZN: AR treated with a combination of catechin (50 mg/kg/day, p.o.) and cetirizine (5 mg/kg, p.o.). *, **, ***: significantly different from the NC group at *p* < 0.05, *p* < 0.01, and *p* < 0.001, respectively. #, ###: significantly different from the AR group at *p* < 0.05 and *p* < 0.001, respectively.

Data in Figure 2 illustrate the effect of ovalbumin-induced AR induction in rats on vascular permeability. Immediately after Evans blue dye injection and starting the nasal PBS perfusion, no significant difference in vascular permeability was observed among the studied groups (time 0 min, Figure 2). However, at the end of the 10 min PBS perfusion, untreated AR rats displayed significantly (*p* < 0.01) higher vascular permeability (2.27 ± 0.29 µg/mL) in comparison with the healthy controls (0.55 ± 0.13 µg/mL). After the 10 min nasal ovalbumin (0.3% *w*/*v*) challenge (time 20 min), the untreated AR animals exhibited a significant increase in vascular permeability in comparison with the normal control group (NC), which is illustrated by the increased dye leakage from nasal lavage. The vascular permeability in these animals peaked after 20 min of starting the nasal ovalbumin challenge (10 min after cessation of the ovalbumin perfusion: time 30, Figure 2) and showed a sustained increase during the rest of the perfusion period in comparison with the control healthy animals. On the other hand, treatment of AR animals with catechin, cetirizine, or the combination of catechin and cetirizine significantly reduced the vascular permeability in these animals when compared with the disease control group (AR) at all the measurement points (10–50 min). Although the results showed no significant difference among all treated groups at all points, only the catechin-treated AR rats showed significantly higher than normal vascular permeability at time points 30 and 40 min. Besides, all treated groups showed significantly higher vascular permeability values at 50 min in comparison with the healthy animals (Figure 2).

### 3.3. Effect of Different Treatment Regimens on Mast Cell and Blood Histamine

**Figure 3:**
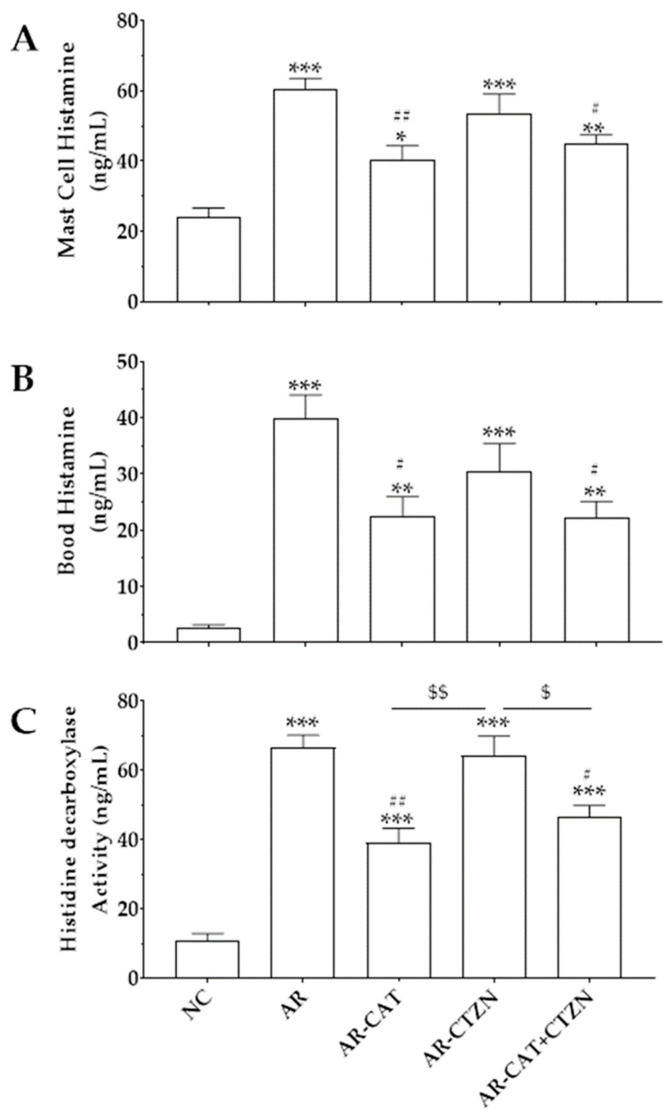
Effect of different treatments on histamine release from mast cells (**A**), blood histamine levels (**B**), and histidine decarboxylase (HDC) activity (**C**). Data represent the mean ± SEM of six animals. NC: normal control, AR: allergic rhinitis disease control, AR-CAT: AR treated with catechin (100 mg/kg/day), AR-CTZN: AR treated with cetirizine (10 mg/kg, p.o.), AR-CAT+CTZN: AR treated with a combination of catechin (50 mg/kg/day, p.o.) and cetirizine (5 mg/kg, p.o.). *, **, ***: significantly different from the NC group at *p* < 0.05, *p* < 0.01, and *p* < 0.001, respectively. #, ##: significantly different from the AR group at *p* < 0.05 and *p* < 0.01, respectively. $, $$: significantly different from the AR-CTZN group at *p* < 0.05 and *p* < 0.01, respectively.

At the end of the 14-day sensitization period, challenging the untreated AR rats with ovalbumin significantly (*p* < 0.001) increased histamine concentrations in the mast cells and blood as compared with the normal control group (Figure 3A,B, respectively). Daily administration of catechin during the sensitization period, either alone (AR-CAT) or combined with cetirizine (AR-CAT+CTZN), significantly decreased the measured histamine concentrations in the cells and blood compared with the untreated AR group. Conversely, animals treated with cetirizine alone (AR-CTZN) did not show any significant decrease in these histamine levels. Noteworthy, the combination regimen (catechin 50 mg/kg + cetirizine 5 mg/kg) was as effective as catechin administration at 100 mg/kg (Figure 3A,B). Besides, all treatment regimens showed significantly higher than normal mast cells and blood histamine levels.

### 3.4. Effect of Different Treatment Regimens on HDC Activity

Ovalbumin sensitization of the untreated animals (AR) significantly increased the activity of HDC as compared with the control healthy group. Treatment of the allergic rats with catechin daily at 100 mg/kg (AR-CAT) or 50 mg/kg (AR-CAT+CTZN) significantly decreased the HDC enzyme activity compared with the AR group. On the other hand, treatment with cetirizine alone did not change HDC activity (Figure 3C).

### 3.5. Effect of Different Treatment Regimens on Barbiturate-Induced Sleeping Time in Balb/c Mice

Data in Table 2 illustrate the effect of various treatment protocols on barbiturate-induced sleeping time in Balb/c mice. Catechin treatment at 100 mg/kg/day for 14 days did not alter the barbiturate-induced sleeping pattern in Balb/c mice compared to the vehicle-treated animals. A single-dose administration of cetirizine at 10 mg/kg significantly, although modestly, increased the barbiturate-induced sleeping time in comparison with the catechin-treated mice. However, the barbiturate-induced sleeping time in animals treated with the combination of catechin (50 mg/kg/day) and cetirizine (5 mg/kg) was comparable to the results in the vehicle-treated mice. None of the treatments altered the sleeping onset (data not shown). These data illustrate that all treatment protocols in the current work, such as the vehicle, lack any measurable sedative effects (Table 2).

### 3.6. Molecular Docking Studies

**Figure 4:**
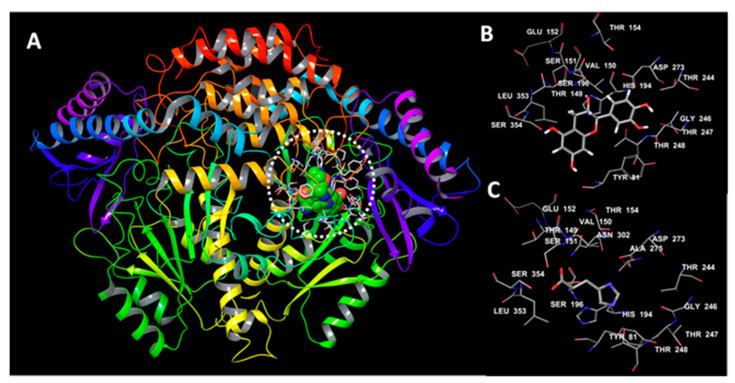
Human histidine decarboxylase with docked compounds. (**A**) The binding site with docked compounds shown in green spheres; (**B**) catechin binding site; and (**C**) *L*-histidine binding site.

The extra-precision docking run was used to analyze the mode of catechin interaction with HDC (Figure 4). Catechin forms a hydrogen bond with the catalytically important residue S354. Catechin, *L*-histidine, and histamine produced docking scores of −8.49, −6.33, and −5.46, respectively. This implies a stronger binding of catechin with the HDC active site compared with the natural substrate; *L*-histidine. Previous research identified S354 as the most critical residue for HDC activity [11]. The overall hydrogen bonding scores (H-bond), the low distance, and the higher total electrostatic attractions might explain the favorable interaction of catechin with S354 (Table 3).

### 3.7. Binding Energy Decomposition

Catechin produced the strongest binding energy across all the tested compounds (Table 4). The binding energy score for catechin was approximately −13 kcal/mol higher than *L*-histidine. According to energy decomposition analysis, the electrostatic interactions (*E_ele_*) were the dominant forces in the binding affinity, with values of −36.83, −35.87, and −24.75 kcal/mol for the catechin–HDC, *L*-histidine–HDC, and histamine–HDC complexes, respectively. Besides, the catechin–HDC showed favorable van der Waals (*E_vdw_*), hydrogen bonds (∆*E_H_*), and lipophilic interactions ∆*E_Lipo_* (Table 4).

## 4. Discussion

In this study, we tested if catechin administration, either alone or combined with the well-established antihistaminic cetirizine, would improve AR in a rat model. We hypothesized that catechin administration, via inhibition of HDC, would decrease the dose of cetirizine, and hence improve tolerance without loss of effectiveness. The results illustrated that chronic catechin (100 mg/kg/day) administration reduced AR clinical scores as the single-dose cetirizine (10 mg/kg). Importantly, when animals received a combination of half the doses of each drug, they showed normalized clinical scores. The current results introduce catechin-induced HDC inhibition, and hence decreased histamine production, as a major mechanism underlying its anti-allergic effects in AR.

The animal models of ovalbumin-induced allergy [23,24,25] reproduce the clinical symptoms of AR such as increased itching, sneezing, and nasal discharge [1,3,4,6,7]. Activation of mast cells in the epithelium and underlying tissues of the nasal cavity by IgE releases various mediators such as histamine, which stimulates afferent nerves [26]. In the present study, challenging the sensitized rats with ovalbumin nasally increased the disease-related clinical scores. Previous research confirmed that nasal hyper-reactivity is a hallmark of AR [4]. Histamine is an itch-producing substance that provokes sneezing, itching, and nasal discharge through activation of neural pathways and induction of vasodilation and vascular permeability when applied locally [5,26,27]. Thus, the H_1_-blocking activity of cetirizine explains the significantly lower clinical scores observed in the cetirizine-treated rats in the current work. These results are supported by the high efficacy of cetirizine in the treatment of human AR, and its consideration as a benchmark for comparator for assessment of AR treatments in clinical studies [28]. Treatment with catechin, which also significantly reduced the AR clinical scores can be attributed to its inhibition of histamine synthesis [17,18,19]. Besides, animals treated with the combination benefited from both H_1_ receptor blockade (cetirizine) and decrease histamine synthesis (catechin) to show normalized clinical scores significantly lower than either drug alone.

Although the pathological processes of allergic reactions are complex, the role of histamine is well established. Mast cells and basophils are major sources of histamine release after provocation [26,29]. Animal models of allergy, including AR, display higher than normal histamine concentrations and responsiveness [12,30]. The results of the current work show that sensitization of rats with ovalbumin for 14 days significantly increases total blood histamine levels and its release from mast cells. Moreover, these animals demonstrated the highest HDC activity. Previous studies showed that an ovalbumin challenge activates the transcription factor NF-κB and the expression of downstream targets that increased histamine levels [23,29,31]. Although cetirizine in the current work did not significantly affect histamine levels in allergic animals, it significantly lowered the AR clinical score in such animals, presumably via inhibition of H_1_-mediated signaling [5,28]. Treatment with catechin, however, showed significantly decreased histamine concentrations in the blood of allergic rats and inhibited its release from mast cells isolated from such animals, which illustrates its role as a potential inhibitor of mast cell HDC [12,17,18,19].

One important pharmacological aspect of histamine is its regulation of vascular function, endothelial barrier, and mucosal inflammation [4,26,32]. Increased signaling by histamine, which impairs vascular permeability, reflects the extent of inflammation in target tissues. The increased expression of H_1_ receptors and N-methyltransferase in the mucosa during AR augments histamine-mediated signaling. Histamine increases allergen absorption by disruption of the epithelial barrier and enhances the expression of the adhesion molecules VCAM-1 and ICAM-1 that favor tissue invasion by inflammatory cells [27,33]. This process is further magnified by the increased production of the chemotactic factors IL-8 and GM-CSF and the increased production of IL-6 which damages the epithelium [26,32,34].

In the current study, we assessed vascular permeability of the nasal mucosa by Evans blue dye leakage in the nasal lavage before and after the ovalbumin challenge. The results of that experiment illustrated a higher-than-normal vascular permeability in all allergic rats. However, the untreated AR rats displayed the highest vascular permeability before and after the ovalbumin challenge. Most importantly, treatment of the AR rats with catechin, cetirizine, or their combination significantly mitigated the ovalbumin challenge-induced increase in vascular permeability. Experimental clinical evidence showed that nasal antigen challenge increases eosinophil infiltration into the nasal mucosa and increases the release of histamine, kinins, leukotrienes, and platelet-activating factor [26,27,35]. Catechin-mediated inhibition of HDC decreases tissue histamine synthesis and is expected to attenuate the allergen-induced vasodilatation. Even though cetirizine does not affect histamine synthesis, its potent H_1_ receptor blocking activity limits the vasodilatory actions of histamine [26,32]. The combination regimen thus shows decreased vascular permeability by the action of two different mechanisms: inhibition of HDC by catechin and H_1_ receptor blockade by cetirizine. The combination regimen, however, did not show better protection than the cetirizine treatment alone against ovalbumin-induced increased vascular permeability in the current study, presumably because these animals received only half the doses of catechin and cetirizine.

The formation of histamine from *L*-histidine requires the catalyst HDC [9,10,36]. In rodents, HDC localizes in the hypothalamic region of the brain, glandular regions of the stomach, and fetal liver. Histamine synthesis by HDC occurs by the decarboxylation of *L*-histidine in the presence of pyridoxal 5′-phosphate—which is the only known histamine generation mechanism in mammalian cells [9,10,11,36]. In this study, the activity of HDC increased in stomach tissues of ovalbumin-induced allergic rats as compared with the normal control animals. Importantly, treatment with catechin abrogated this augmented activity of the enzyme. HDC has a highly restrictive and hydrophobic catalytic site [37]. It only binds to histidine or imidazole containing analogs such as α-fluoromethyl histidine, histidine methyl ester, and natural polyphenols such as catechins by inducing a similar conformational change of the enzyme-bound substances [18,38]. Importantly, patients with allergic diseases [39,40] illustrate high concentrations of HDC, akin to the increased expression and activity of HDC evident in experimental allergy models [12,41] and in the current work. Moreover, the administration of H_1_ blockers proved effective in suppressing allergen-induced upregulation of HDC in rats [41]. Thus, the neoteric regimen of catechin (5 mg/kg/day) plus the anti-histaminic drug cetirizine (5 mg/kg) has demonstrated worthier efficacy by virtue of the combined inhibition of HDC and blockade of H_1_ receptors.

To further confirm the HDC-inhibitory effects of catechin, a docking run followed by binding energy estimation were carried out in the current work to conclude the binding strength and the forces contributing to catechin recognition by HDC. To benchmark the obtained data for catechin, we compared the obtained results with a standard known inhibitor, histidine methyl ester. Histidine methyl ester is a strong HDC inhibitor with a *Ki* value equal to 0.46 µM [42]. Compared with histidine methyl ester, catechin showed improved docking scores and higher binding free energy. The favorable binding parameters with comparable efficiency to histidine methyl ester support the binding strength of catechin with HDC with expected inhibition comparable with histidine methyl ester.

In further clarifying the potential combination of catechin and cetirizine, we estimated and compared their binding potencies and their contributing forces (Table 3 and Table 4). Cetirizine showed a high binding score of −9.4 which is slightly higher than catechin (−8.49). In addition, the binding energy was almost similar for both catechin and cetirizine (Table 3). However, these higher binding strength parameters were not associated with interaction with the S354 which is critical for enzyme activity [11]. Therefore, we suggest that cetirizine might bind with HDC with good potency and lower catalytic efficiency due to its inability to bind with S354 (Supplementary Figure S2). This is further confirmed by the zero H-bond score and zero value for hydrogen bonds with S354 (Table 3). Based on these data, we suggest that both catechin and cetirizine can bind with HDC, but catechin is a more effective inhibitor due to interference with the catalytic residue function. Furthermore, the action of cetirizine might be additive to the action of catechin. In a similar study, the combined treatment with catechin and cetirizine decreased the HDC activity [12].

We evaluated the sedative effects of catechin as well as cetirizine at the doses used in the current study by their effect on the barbiturate-induced sleeping time in mice. This method is widely employed for CNS safety pharmacological studies [43,44]. The results illustrated no change in the barbiturate-induced sleeping time in treated rats compared to the vehicle-treated animals. Interestingly, the cetirizine-treated rats slept a little longer than the catechin-treated ones—but the increases in sleeping time were, however, too minimal to show any superiority of catechin over cetirizine in this regard. Altogether, these data show that catechin and cetirizine at the doses used in the current study, or their probable metabolites, lack appreciable sedative effects. One limitation of the current study is that we did not evaluate central histamine levels or the ability of catechin to reach the CNS at pharmacological levels. In addition, other possible adverse effects of HDC inhibition in different tissues will be dependent on the importance of histamine signaling to these respective organs, which was not the focus of the current study.

Catechin and its natural derivatives are HDC inhibitors that have shown effectiveness in allergic disorders in diverse studies [17,18,19,31,45]. Catechin acts to inhibit the conversion of the amino acid *L*-histidine to histamine by HDC. In the current study, the stomachs of catechin-treated allergic rats displayed lower HDC enzyme activity than the cetirizine-treated or untreated counterparts. Histamine is one of the inflammatory mediators which produces various symptoms of allergy such as itching, sneezing, spasm of the airways, and tissue swelling [5,7,13]. Henceforth, targeting the HDC enzyme is a plausible anti-allergic mechanism. Cetirizine, by blocking H_1_ is an excellent drug in allergic conditions [14,15]. However, the adverse effects of cetirizine, including its sedative effects and change in the quality of sleep, are important limitations [16,46]. Catechin, besides the lack of sedative effects as shown in the current study, has other antioxidant and pleiotropic health effects [47,48].

## 5. Conclusions

The results of the current study show that a combination of catechin and cetirizine is beneficial in the management of AR. The results suggest that catechin has significant anti-allergic activity in experimental AR. More importantly, the neoteric combination of catechin and cetirizine showed promising anti-allergic activity in these animals, given the fact that half the dose of each drug was used. The neoteric combination is more effective than either treatment alone in reducing the clinical symptoms without increasing adverse effects, but as effective in decreasing vascular permeability. The neoteric combination works by inhibition of HDC by catechin, added to the anti-histaminic activity of cetirizine.

## Figures and Tables

**Table 1:** Clinical scoring criteria for allergic rhinitis.

Feature	Characteristic	Clinical Scoring
Nasal itch	None	0
	Scratching nose lightly one to two times	1
	Scratching the nose and face constantly	2
Sneeze	None	0
	One to three times	1
	Four to ten times	2
	Eleven or more times	3
Nasal discharge	None	0
	Secretions flow to anterior nostril	1
	Secretions surpass anterior nostril	2
	Secretions cover the face	3

**Table 2:** Effect of different treatment regimens on barbiturate-induced sleeping time.

Parameter	Vehicle	CAT	CTZN	CAT+CTZN
Barbiturate-induced sleeping time (min)	31.33 ± 1.66	29.66 ± 0.76	34.66 ± 1.02 *	30.66 ± 0.4

Data represent the mean ± SEM of six animals and are analyzed with one-way ANOVA followed by Tukey’s test. * Denotes significant difference from the catechin (CAT) group (*p* < 0.05). CTZN: cetirizine.

**Table 3:** The docking scores and binding with S354 for the compounds binding with histidine decarboxylase.

Compound	XP Docking Score	H-Bond	Elec	S354 H-Bond	S354 Distance
Catechin	−8.49	−3.25	−39.09	−0.47	2.9
*L*-histidine	−6.33	−3.6	−25.57	−1	2.7
Histamine	−5.46	−1.44	−20.88	−0.1	2.2
Cetirizine	−9.4	0	−8.9	0	2.7
Histidine methyl ester	−5.29	−1	−16.01	−1	1.7

**Table 4:** Molecular mechanics–generalized Born surface area (MM/GBSA) binding energies (kcal/mol) for the drugs binding with histidine decarboxylase.

Compound	Binding Energy	∆*E_elec_*	∆*E_vdw_*	∆*E_H_*	∆*E_Lipo_*	∆*E_solv_*
Catechin	−50.86	−36.83	−28.22	−1.60	−28.10	39.09
*L*-histidine	−37.64	−35.87	−18.28	−2.55	−10.19	25.57
Histamine	−32.27	−24.75	−16.00	−0.81	−11.74	20.88
Cetirizine	−49.36	−13.19	−15.25	−1	−59.5	25.53
Histidine methyl ester	−51.1	−46.13	−23.67	−2.56	−12.82	31.03

## Data Availability

Data are contained within the article or available upon reasonable request from the corresponding author.

## Supplementary Materials

The following supporting information can be downloaded at: https://www.mdpi.com/article/10.3390/life12060820/s1, Figure S1: The generated grid box (violet box) contains the excluded volume occupied by pyridoxal-5′-phosphate (colored spheres); Figure S2: The docking site of cetirizine with histidine decarboxylase. No hydrogen bonding can be formed with S354.
