# Supplementary figures and images for: Ameliorative Effect of a Neoteric Regimen of Catechin plus Cetirizine on Ovalbumin-Induced Allergic Rhinitis in Rats
Source: Life (Basel). 2022 May 31;12(6):820. doi: 10.3390/life12060820 (PMC9225010; doi:10.3390/life12060820)

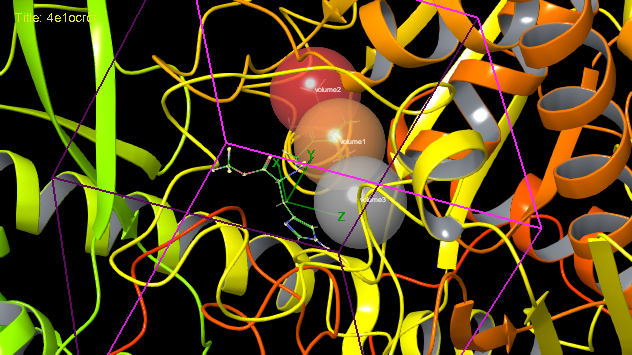

Supplement: Supplementary file 1 [file life-12-00820-s001.zip › Supplementary Figure S1.png]

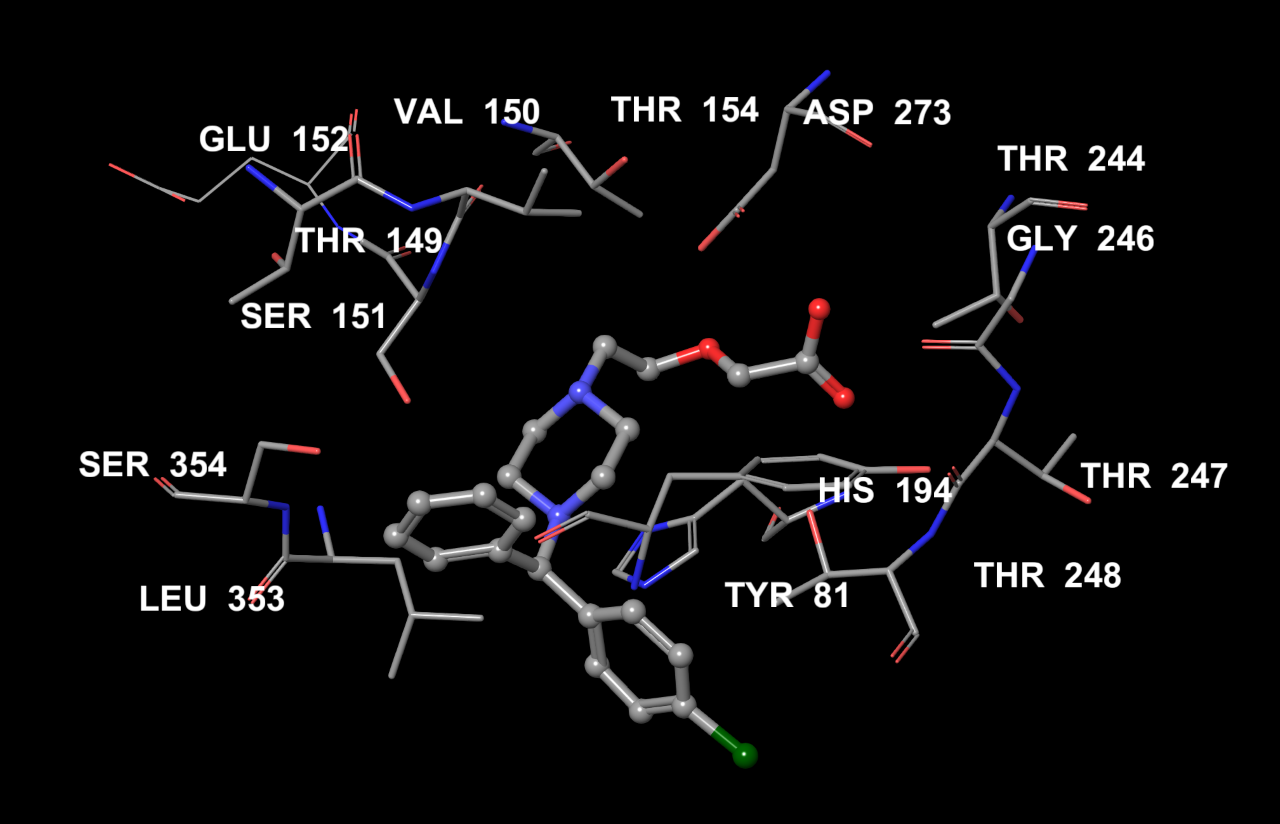

Supplement: Supplementary file 1 [file life-12-00820-s001.zip › Supplementary Figure S2.png]
